# P-1779. A tale of two drugs: differential effects of sickle cell therapies on Plasmodium falciparum growth

**DOI:** 10.1093/ofid/ofaf695.1948

**Published:** 2026-01-11

**Authors:** Sesh A Sundararaman, Ellora Daley, Tonimarie Ruggiero, Osheiza Abdulmalik, Audrey John

**Affiliations:** Children's Hospital of Philadelphia, Philadelphia, PA; Children’s Hospital of Philadelphia, Philadelphia, Pennsylvania; Children’s Hospital of Philadelphia, Philadelphia, Pennsylvania; Children’s Hospital of Philadelphia, Philadelphia, Pennsylvania; Children's Hospital of Philadelphia, Philadelphia, PA

## Abstract

**Background:**

Malaria is a major contributor to the morbidity and mortality of sickle cell anemia (SCA) in Africa. However, patients with SCA are protected against some manifestations of severe malaria including hyperparasitemia and cerebral malaria. This protection is thought to result from the inhibition of parasite growth in the setting of red cell sickling. Novel SCA therapies that decrease red cell sickling are currently in clinical trials. The development of these therapies raises important questions about how SCA treatments modulate malaria infection and risk of severe disease.

**Methods:**

Using dose response and longitudinal growth assays, we examine the effects of two sickle cell therapies: hydroxyurea (HU), and hemoglobin polymerization inhibitors (voxoletor and osivelator) on *Plasmodium falciparum* growth in sickle cell (HbSS) and non-sickle cell (HbAA) red blood cells *in vitro*.

**Results:**

HU inhibited *P. falciparum* growth at concentrations that are typically achieved in patients receiving daily therapy. However, parasites developed partial resistance to HU treatment under continuous culture. While the hemoglobin polymerization inhibitors, voxoletor and osivelator, also inhibited parasite growth, this inhibition was only observed at supertherapeutic concentrations. Moreover, treatment of HbSS red blood cells with therapeutic concentrations of either voxoletor or osivelator significantly increased parasite growth under hypoxic conditions.

**Conclusion:**

Our findings highlight the importance of considering risks of infection associated with therapies for SCA. Malaria chemoprevention could be considered as an adjunctive therapy in clinical trials of hemoglobin polymerization inhibitors in malaria endemic countries. While HU therapy has been shown to decrease the risk of malaria in clinical trials in Africa, the development of HU resistance in *P. falciparum* could offset these gains. Longitudinal studies are necessary to determine if resistance can develop in real world settings, and how this might affect the incidence and severity of malaria in patients on HU therapy. Future studies will also examine the mechanisms *P. falciparum* growth inhibition and resistance.

**Disclosures:**

All Authors: No reported disclosures

